# Goliath, meet David

**DOI:** 10.1038/s44319-024-00257-9

**Published:** 2024-09-16

**Authors:** K Heran Darwin

**Affiliations:** New York University Grossman School of Medicine, New York, NY USA

**Keywords:** Economics, Law & Politics, Microbiology, Virology & Host Pathogen Interaction, Pharmacology & Drug Discovery

## Abstract

Will the battle against microbes ever end?

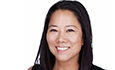

There is an oft-repeated quote, allegedly by a US Surgeon General in the 1970s: “It’s time to close the book on infectious diseases … and shift national resources to such chronic problems as cancer and heart disease”. I cannot find a reliable source for it but if these words were really ever uttered, talk about getting it so wrong.

Microbes are the most adaptable organisms on the planet. With doubling times often less than an hour, it is easy to see how many bacteria—and parasites and viruses—can adjust to new conditions faster than their hosts can evolve to evade them. Even the mammalian immune system—evolved and fine-honed over millennia to squash microbial invaders—is not always able to deal with a bacterial infection. Humans have taken the conflict with pathogens a step further by developing vaccines and antibiotics, but microbes always find a way: to quote François Jacob, “The dream of every cell is to become two cells”.

Without any doubt, antibiotics have been one of the greatest inventions of humankind and have saved millions, if not billions, of lives from death or disease. Unfortunately, their improper use in settings from agriculture to clinics has selected for bacteria that have become increasingly resistant against nearly everything in our antibiotics arsenal. For example, if a patient is prescribed a course of antibiotics to treat a particular bacterial infection, they must complete the drug regimen to minimize the emergence of a rare mutant bacillus that is resistant to the drug. Antibiotics reduce bacterial burden and give the host immune system a chance to finish the job: if a pathogen is not cleared, a resistant clone can expand, making it not only harder to eliminate, but also risks infecting new hosts in the community. This scenario, unfortunately, has become a reality: “ESKAPE” is an acronym describing bacterial genera (*Enterococcus*, *Staphylococcus*, *Klebsiella*, *Acinetobacter*, *Pseudomonas*, and *Enterobacter*) with pathogenic species that have become so drug-resistant that they have been designated a One Health problem (Miller and Arias, 2024).

Antibiotic resistance is usually conferred by specific enzymes or transporters encoded in discreet DNA sequences. Bacteria can also become drug-resistant by acquiring mutations that alter the drug target itself. Worryingly, bacteria can transfer these resistance traits amongst themselves using phages, transformation, or conjugation. Complicating the situation even more, some pathogenic bacterial strains may not necessarily have become more drug-tolerant or resistant but have just become more prevalent. This phenomenon suggests that there are other factors at work in the environment or host population that select these pathogens. Whatever the causes, new ways to treat various bacterial diseases are desperately needed.

While the goal to find new ways to kill bacteria seems straightforward, biology is never so simple. It is increasingly evident that interactions with microbes during childhood may protect us from various insults, from allergies to autoimmunity to obesity. Our gut microbiota also provides essential vitamins and nutrients and protects us against pathogens. Thus, simply eliminating all bacteria from our bodies would affect a much larger number of beneficial commensals. In addition to minimizing the impact on our microbiota, good antimicrobials should avoid turning normally harmless bacteria into pathogens. For example, some antibiotics can trigger the secretion of deadly toxins by some *Escherichia coli* strains or allow *Clostridium difficile* to bloom and cause severe intestinal distress.

Pertinent to this topic is the idea that the term “pathogen” should be kicked to the curb. For years, Arturo Casadevall and Liise-anne Pirofski have asserted that one’s symbiont can be another person’s pathogen, just based on the physiologic status of the host; simply put, “a microbe cannot cause disease without a host (Casadevall and Pirofski, 2014)”. Moreover, one animal species’ commensal bacterium can be lethal for another. Famous examples are *Salmonella* and *Campylobacter* species that often colonize chickens asymptomatically, but can cause severe diseases in humans who ingest these bacteria. In an extreme example, normally harmless bakers’ yeast can kill someone if they are severely immunocompromised.

Given the immediate need for new ways to treat old infections such as *Mycobacterium tuberculosis* and rapidly emerging ESKAPE species, many organizations are pushing for new drugs to get to the clinic. Ideally, these new drugs would precision-bomb pathogens to minimize killing beneficial microbes, a tall order to be fair.

My impression of drug development is that it is complicated and expensive. I am not involved in developing antibiotics, but I was on a scientific advisory board that attempted to bring the public and private sectors together to find new ways to treat tuberculosis (TB). This alliance was exciting to me; perhaps small molecules that did not work in other clinical contexts could work for TB. The intent was commendable, but the execution left a lot to be desired. In one instance, a group of researchers had performed a screen for small molecules that could specifically kill bacteria in macrophages, without killing the macrophages themselves. These compounds could then be further tested in small animal models for host toxicity and effectiveness against controlling bacterial growth. However, I also witnessed medicinal chemist consultants get bogged down in the minutia of pharmacokinetics rather than embrace a compound’s ability to kill bacteria without killing host cells. Most frustratingly, I listened to how a compound finally emerged after years of work and survived the gauntlet of toxicity tests required for getting it to treat infected animals, only to learn that it was unclear who would pay for the experiment. At this point, I got exasperated and asked, “What are we doing here?” I never got an answer because the project ended with the onset of the COVID-19 pandemic before any tests could be done.

Another aspect of antimicrobial discovery that confuses me is the need to know the ‘mechanism of action’ (MOA) of new drugs, which the FDA officially does *not* require, yet when I speak with certain agencies, there is an insistence on it. And yet, the MOAs for current frontline TB drugs are largely unknown, and these drugs were introduced into humans long before we had the tools to understand how they worked. When did we become beholden to MOA? Don’t get me wrong, knowing a drug’s target is a great idea, mainly so that new drugs could be developed that use the same target, as well as gain new knowledge about critical bacterial processes. But if more than 4000 people are dying of TB every day, should we let patients wait for the MOA of a potential drug that has already passed safety tests?

Microbes have been on our planet long before we existed. Humanity will always be playing Whac-A-Mole with infectious diseases, so we must constantly remain vigilant. While cancer and heart disease should remain top priorities, infectious diseases should never be relegated to closed-book status.

## Supplementary information

Peer Review File
