# Supplementary material for: Goliath, meet David
Source: EMBO Rep. 2024 Sep 16;25(10):4091–2. doi: 10.1038/s44319-024-00257-9 (PMC11467124; doi:10.1038/s44319-024-00257-9)
Supplement: Supplementary file 1 — Peer Review File [file 44319_2024_257_MOESM1_ESM.pdf]

EMBO reports

# Goliath, meet David

Heran Darwin

*Corresponding author(s): Heran Darwin (Heran.Darwin@med.nyu.edu)*

**Review Timeline:**

| | |
|---|---|
| Submission Date: | 5th Sep 24 |
| Accepted: | 5th Sep 24 |

*Editor: Holger Breithaupt*

## Transaction Report: This article type is not peer reviewed.
